# Factors That Influence Mortality in Critically Ill Patients with SARS-CoV-2 Infection: A Multicenter Study in the Kingdom of Saudi Arabia

**DOI:** 10.3390/healthcare9121608

**Published:** 2021-11-23

**Authors:** Khalid A Alhasan, Mohamed A Shalaby, Mohamad-Hani Temsah, Fadi Aljamaan, Reem Shagal, Talal AlFaadhel, Mohammed Alomi, Khalid AlMatham, Adi J. AlHerbish, Rupesh Raina, Sidharth K. Sethi, Sarah Alsubaie, Marwah H Hakami, Najla M Alharbi, Razan A Shebeli, Hanan Mohamed Nur, Ohoud F Kashari, Faiza A Qari, Amr S Albanna, Jameela A Kari

**Affiliations:** 1 Pediatrics Department, College of Medicine, King Saud University, Riyadh 11451, Saudi Arabia; kalhasan@ksu.edu.sa (K.A.A.); mtemsah@ksu.edu.sa (M.-H.T.); rshagal@ksu.edu.sa (R.S.); aalherbish@KSU.EDU.SA (A.J.A.); salsubaie@KSU.EDU.SA (S.A.); 2 Pediatric Nephrology Center of Excellence, Department of Pediatrics, King Abdulaziz University, Jeddah 21589, Saudi Arabia; dr.m.a.shalaby@gmail.com; 3 Department of Pediatrics, King Abdulaziz University Hospital, Jeddah 21589, Saudi Arabia; 4 Critical Care Department, College of Medicine, King Saud University, Riyadh 11451, Saudi Arabia; faljamaan@ksu.edu.sa; 5 Department of Medicine, College of Medicine, King Saud University, Riyadh 11451, Saudi Arabia; tfaadhel@KSU.EDU.SA; 6 Prince Mohammed bin Abdulaziz Hospital, King Salman Center for Kidney Diseases, Ministry of Health, Riyadh 14214, Saudi Arabia; malomi@moh.gov.sa; 7 King Fahad Medical City, Ministry of Health, Riyadh 11525, Saudi Arabia; Kalmatham@kfmc.med.sa; 8 Pediatrics Nephrology, Akron Children’s Hospital, Akron, OH 44241, USA; rraina@akronchildrens.org; 9 Akron Nephrology Associates, Cleveland Clinic Akron General, Akron, OH 44241, USA; 10 Pediatric Nephrology, Medanta, The Medicity, Gurgaon 122001, India; sidsdoc@gmail.com; 11 Pediatric Department, East Jeddah General Hospital, Jeddah 636012, Saudi Arabia; marwahakami@outlook.com (M.H.H.); Najla2400@hotmail.com (N.M.A.); razan.shebelli@gmail.com (R.A.S.); Boomerang.5678@gmail.com (H.M.N.); Dr.o.kashari@gmail.com (O.F.K.); 12 Department of Medicine, College of Medicine, King Abdulaziz University, Jeddah 21589, Saudi Arabia; faizaqari@gmail.com; 13 King Abdullah International Medical Research Center, King Saud Bin Abdulaziz University for Health Sciences, Jeddah 11481, Saudi Arabia; amralbanna@gmail.com

**Keywords:** COVID-19 disease, outcomes, SARS-CoV-2 infection, risk of mortality, Saudi Arabia

## Abstract

Background: SARS-CoV-2 infection has a high mortality rate and continues to be a global threat, which warrants the identification of all mortality risk factors in critically ill patients. Methods: This is a retrospective multicenter cohort study conducted in five hospitals in the Kingdom of Saudi Arabia (KSA). We enrolled patients with confirmed SARS-COV-2 infection admitted to any of the intensive care units from the five hospitals between March 2020 and July 2020, corresponding to the peak of recorded COVID-19 cases in the KSA. Results: In total, 229 critically ill patients with confirmed SARS-CoV-2 infection were included in the study. The presenting symptoms and signs of patients who died during hospitalization were not significantly different from those observed among patients who survived. The baseline comorbidities that were significantly associated with in-hospital mortality were diabetes (62% vs. 48% among patients who died and survived (*p* = 0.046)), underlying cardiac disease (38% vs. 19% (*p* = 0.001)), and underlying kidney disease (32% vs. 12% (*p* < 0.001)). Conclusion: In our cohort, the baseline comorbidities that were significantly associated with in-hospital mortality were diabetes, underlying cardiac disease, and underlying kidney disease. Additionally, the factors that independently influenced mortality among critically ill COVID-19 patients were high Activated Partial Thromboplastin Time (aPTT )and international normalization ratio (INR), acidosis, and high ferritin.

## 1. Introduction

Coronavirus disease (COVID)-2019 is caused by severe acute respiratory syndrome coronavirus 2 (SARS-CoV-2) [1,2], a novel coronavirus that emerged in December 2019 in Wuhan, China [2]. On 30 January 2020, the World Health Organization declared the COVID-19 outbreak a Public Health Emergency of International Concern and, on 11 March 2020, as a global pandemic. Worldwide, more than 196 million people were infected by the end of July 2021, with more than 4.2 million deaths [3]. The first country of the Gulf Council countries (GCC) to report cases was the UAE, which was linked to a recent visit to Wuhan, China; later, other countries of the GCC reported their first cases, which were linked to a visit to Iran. Saudi Arabia was the last country of the GCC to report its first case, which was diagnosed on 2 March 2020 [4,5] and was also linked to a visit to Iran, in spite of the efforts taken by the Saudi local authorities to keep the disease outside of the country by suspending the Umrah (minor pilgrimage to Mecca required at least once in the lifetime of Muslims that can be undertaken at any time of the year) and tourism visas and some international flights, especially to areas where the disease was reported, as soon as the disease was declared. Since reporting the first Saudi case, the local authorities made fast stepwise decisions of the suspension of school attendance, closure of restaurants, suspension of all international flights, and finally, the complete lockdown of all the major cities, which lasted for almost two months. The recent statistics showed that 525,730 confirmed cases and 8237 deaths were reported as of the end of July 2021 in Saudi Arabia [6]. 

Given the high contagiousness of the virus, especially with the emergence of highly transmissible variants such as the Delta variant; the presence of asymptomatic and subclinical cases, especially among the vaccinated population; and significant differences in susceptibility to COVID-19 morbidity and mortality among the different age groups and types of population, knowledge on COVID-19 pathophysiology, management, complications, and the underlying risk factors for mortality is a top priority for researchers in order to help health care providers to direct their resources and deliver care according to the predicted outcomes and take the most appropriate measures to improve clinical outcomes among the different types of patient populations. Studies that addressed recovery and mortality from COVID-19 disease have shown wide variability worldwide [7]. 

Multiple studies have addressed clinical outcomes in COVID-19 patients: many of them have shown a correlation of mortality with advanced age and male gender; some have shown an association with smoking and certain chronic comorbidities, such as underlying diabetes mellitus, chronic renal insufficiency, chronic obstructive lung disease, and cardiovascular diseases; some have addressed clinical parameters, such as the severity of respiratory failure [8] and acute renal failure; and others have addressed laboratory investigations as predictors of outcome and found significant correlations with certain inflammatory markers, such as high-sensitivity C-reactive protein, D dimer, and procalcitonin [9,10,11,12].

The prevalence rate of COVID-19 among the Saudi population was 6.1%, corresponding to an incidence of 879.7 per 100,000 population and a case fatality of 2.0% between March and August 2020. Advanced age, male gender, hypoxia on presentation, underlying cardiovascular disease, and malignancy were highly associated with mortality as per one of the previous largest studies [13]. Most of the previous large national studies have shown that the median age of the affected population was between 35 and 45 years, and males were affected more than females [13,14]. Certain studies have addressed special populations, such as the elderly, or those with severe disease, such as patients on mechanical ventilation, or as part of the RCT of an interventional study [15,16].

This urged us to conduct this multicenter retrospective cohort study to identify the factors associated with mortality in a group of patients that was characterized to have worse outcomes, critically ill patients, who were admitted to all five participating intensive care units (ICUs) in the KSA.

## 2. Methods

### 2.1. Study Design and Setting

This retrospective cohort study was performed in five hospitals and health institutions in the KSA (King Abdulaziz University Hospital, King Saud University Medical City, East Jeddah Hospital, King Fahd Medical City, and Prince Mohammed bin Abdulaziz Hospital), all of which are specialist centers that receive patients with a confirmed diagnosis of COVID-19. The need for consent was waived, as this was retrospective and anonymous study.

### 2.2. Sample Selection and Subjects

We enrolled all patients with confirmed SARS-COV-2 infection admitted to any of the ICUs in these hospitals between 20 March 2020 and 27 July 2020, which was the time when the peak number of COVID-19 cases was recorded in the KSA. Patients for whom significant data were missing were excluded. All participating institutions used the same visual triage checklist for acute respiratory infection in suspected cases. The diagnosis of SARS-COV-2 infection was made using real-time reverse transcription polymerase chain reaction analysis of nasopharyngeal secretions, sputum, or endotracheal aspirate. 

### 2.3. Data Source and Instruments

We included all data from paper and electronic health records that were deemed to be clinically relevant based on the published literature and expert opinion. The dataset consisted of demographic data (age, sex, ethnicity, weight, height, and body mass index); presenting symptoms and signs; history of recent contact with a confirmed positive case of COVID-19; initial triage and COVID-19 score at presentation; initial physiological status and hospital course prior to ICU admission; findings on chest radiography; need for and duration of noninvasive or invasive mechanical ventilation; evidence of electrocardiographic abnormalities; evidence of infection; inflammatory markers, including leukocytosis or leukopenia, neutropenia, lymphopenia, high C-reactive protein, troponin, and ferritin; disturbance of the coagulation profile; and details of medications received.

We also recorded comorbidities, namely, diabetes, hypertension, asthma, renal impairment, cardiac disease, hematological or oncological disease, and post-transplant immunosuppression. The initial physiological status and its impact on the outcome was assessed using Sequential Organ Failure Assessment [17] and COVID-19 scores.

Renal function was assessed by the baseline creatinine level, baseline estimated glomerular filtration rate (eGFR), highest creatinine level, and lowest eGFR recorded during the initial 7 days of admission. We also calculated the urine output. The eGFR was calculated using the modified Schwartz formula [18]. The baseline creatinine level was defined as the last value recorded within the 6 months prior to admission to the ICU. For patients admitted for the first time, we used the average GFR based on age, sex, and height [19]. The primary outcome was in-hospital mortality.

### 2.4. Statistical Analysis

Dichotomous variables are summarized as the proportion and continuous variables as the mean. The proportion and 95% confidence interval (CI) of in-hospital mortality in patients admitted to the critical care unit with confirmed COVID-19 were estimated. Factors associated with in-hospital mortality were determined using the Chi-square test. Age, sex, and other variables for which significant associations were found were entered into a multivariable logistic regression model to determine the factors that were independently associated with mortality. All statistical analyses were performed using Stata statistical software (Release 12; StataCorp LP, College Station, TX, USA). Statistical significance was determined by a *p*-value of 0.05 and the 95% CI.

## 3. Results

In total, 229 critically ill patients with confirmed COVID-19 infection were included in the study. Baseline demographic and disease characteristics are shown in Table 1.

**Figure 1:**
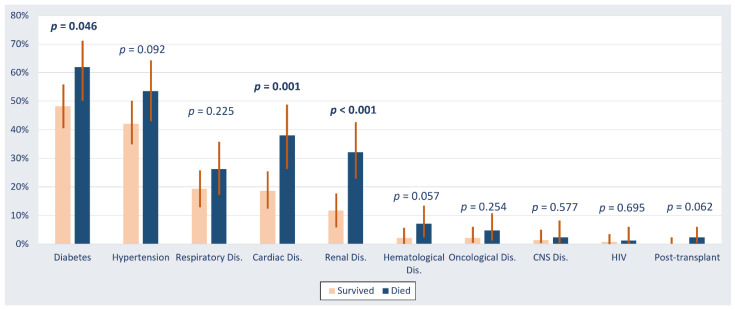
Baseline comorbidities in critically ill patients with COVID-19 stratified by survival outcome. Abbreviations: CNS, central nervous system; Dis., disease; HIV, human immunodeficiency virus.

**Figure 2:**
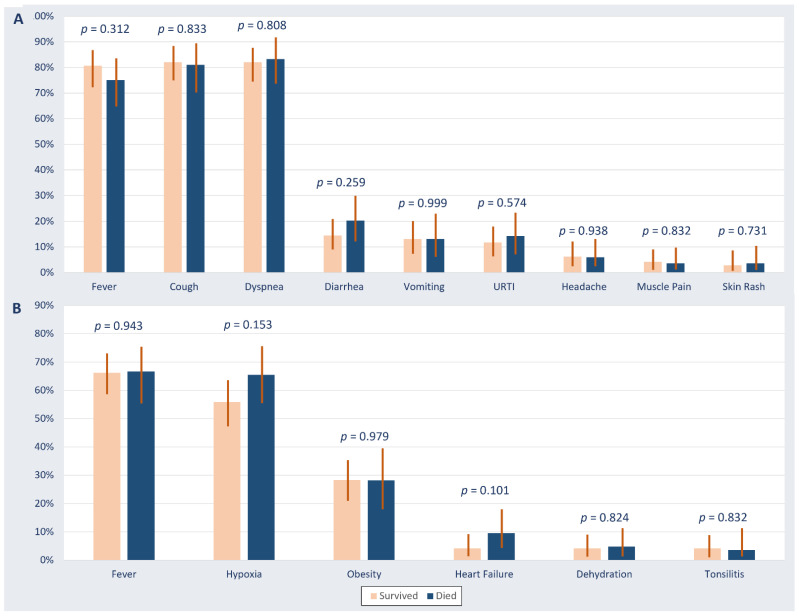
Presenting symptoms (**A**) and signs (B) in critically ill patients with COVID-19 stratified by survival outcome. Abbreviations: URTI, upper respiratory tract infection.

The incidence of in-hospital mortality was 37% (95% CI: 30–43%). The presenting symptoms and signs of patients who died during hospitalization were not significantly different from those observed among patients who survived (Figure 1). The baseline comorbidities that were significantly associated with in-hospital mortality were diabetes (62% vs. 48% among patients who died and survived (*p* = 0.046)), underlying cardiac disease (38% vs. 19% (*p* = 0.001)), and underlying kidney disease (32% vs. 12% (*p* < 0.001)) (Figure 2). The mortality was comparable between Saudi patients (39%) and non-Saudi patients (36%) *p* = 0.670.

Clinical investigations that were significantly associated with mortality included acute deterioration of renal function (91% vs. 52% (*p* < 0.001)), hypernatremia (70% vs. 36% (*p* < 0.001)), hyperkalemia (73% vs. 38% (*p* < 0.001)), anemia (71% vs. 38% (*p* < 0.001)), leukocytosis (92% vs. 67% (*p* < 0.001)), thrombocytopenia (69% vs. 25 (*p* < 0.001)), high APTT (96% vs. 71% (*p* < 0.001)), high INR (64% vs. 26% (*p* < 0.001)), acidosis (85% vs. 49% (*p* < 0.001)), high troponin (85% vs. 49% (*p* < 0.001)), and high ferritin (87% vs. 27% (*p* < 0.001)) (Table 2).

Based on the multivariable regression model, the only factors that independently influenced mortality among critically ill COVID-19 patients were high APTT (adjusted OR: 7.4 (95% CI: 1.2–47.9; *p* = 0.035)), high INR (adjusted OR: 4.1 (95% CI: 1.2–13.8; *p* = 0.025)), acidosis (adjusted OR: 3.7 (95% CI: 1.0–13.8; *p* = 0.047)), and high ferritin (adjusted OR: 14.4 (95% CI: 4.2–49.9; *p* < 0.001)) (Table 3).

## 4. Discussion

In this study, the in-hospital mortality rate was 37% in patients with COVID-19 who required admission to an ICU in the KSA. Diabetes, cardiac disease, and underlying kidney disease were associated with increased mortality. A meta-analysis of 24 studies that included 10,150 patients and was published in 2020 found a mortality rate of 41.6% in those admitted to an ICU [20]. In another multicenter, prospective, observational cohort study that included 64 hospitals in ten African countries (Egypt, Ethiopia, Ghana, Kenya, Libya, Malawi, Mozambique, Niger, Nigeria, and South Africa), the mortality rate was 48.2% in adults with suspected or confirmed COVID-19 infection who were referred to an ICU or high-dependency unit [21]. A higher mortality rate of 60.4% was reported in critically ill patients with COVID-19 in a prospective multicenter cohort study in Libya [22], and a lower rate of 26.2% was reported in a national cohort study in Canada [23]. Therefore, there is a wide variation in mortality in previous reports; however, the rate is broadly consistent across the world. As the pandemic has progressed, the reported mortality rates have decreased from more than 50% to close to 40% [20]. The pooled hospital mortality rate is much lower, as reported in a recent meta-analysis of 80 studies (14%) [24]. Furthermore, lower incidence rates of 7.7% and 3.2% were reported in earlier meta-analyses [25,26], which could be explained by variation in the severity of COVID-19 in the patients included in the studies analyzed. The lower reported cumulative cases and mortality rate in our study could also reflect the overall strict mitigation measures applied by Saudi authorities since the diagnosis of early cases of COVID-19 on 2 March 2020. These measures included a travel ban, the closure of nonessential shops, the suspension of religious activities, school closure, and curfews. Such measures helped to slow down the transmission and avoided the overburden of healthcare systems compared to other countries with advanced healthcare infrastructure [27].

Our finding that diabetes, underlying cardiac disease, and underlying kidney disease were risk factors for mortality in critically ill patients with COVID-19 is similar to that in a meta-analysis of 48 studies, which identified diabetes, cardiovascular disease, renal disease, respiratory disease, malignancy, hypertension, older age, and smoking to be associated with mortality [28]. We also found a higher likelihood of mortality in older patients. Similarly, in another meta-analysis of nine studies, advanced age and hypertension were found to be associated with mortality in adult patients with COVID-19 admitted to an ICU [29]. In a further meta-analysis of patients hospitalized with COVID-19, older age, male sex, hypertension, diabetes, chronic respiratory disease, chronic heart disease, and cardiovascular disease were associated with a higher risk of death [24]. Hypertension was more common in our nonsurvivors than in our survivors, but the difference was not statistically significant. Rates of respiratory disease and malignancy were similar in both our study groups.

We also found that hypernatremia was associated with mortality. This is in line with previous reports showing that dysnatremia (hypernatremia or hyponatremia) is a risk factor for mortality in patients with COVID-19 [30,31]. The dynamics of sodium are an important indicator of the severity of COVID-19, and dysnatremia is reportedly common and associated with a longer hospital stay and a higher risk of death [31]. We also identified hyperkalemia to be a risk factor for mortality. Previous studies have found that both high and low [32] potassium levels are risk factors for mortality. In our study, acidosis was also observed to be a risk factor for death, which is similar to previous reports [31,32]. Other laboratory findings that were associated with mortality (anemia, leukocytosis, thrombocytopenia, high aPTT, high INR, high troponin, and high ferritin) have also been reported to be risk factors for death in patients with COVID-19 [33,34].

Acute kidney injury (AKI) with the rapid deterioration of renal function was significantly more common in nonsurvivors in our cohort and has already been found to be a risk factor for mortality in patients hospitalized with COVID-19 [19]. Gutiérrez-Abejón et al. reported a doubling of the mortality rate in patients with COVID-19 and AKI [34]. Other studies have also demonstrated that AKI is associated with an increased risk of death [35,36]. Patients with a high creatinine level and a low GFR at admission to the ICU had worse outcomes in our study. Kidney impairment is common in patients admitted to an ICU with COVID-19 and is associated with high mortality and a long-term impact on renal function after discharge from critical care [37]. COVID-19 is associated with high rates of AKI, which are not fully explained by known risk factors [38].

Acute myocardial injury and complications were also more common in nonsurvivors in this study. Acute cardiac injury, as defined by troponin elevation, occurs in approximately 16% of patients with COVID-19 and is associated with increased mortality and a prolonged length of stay [38,39,40].

This study has some limitations, in particular, its retrospective design and small study population, which means the result of the study was not powered to show the causality on general population. However, it was a multicenter study and examined the risk factors for mortality in patients with COVID-19 in detail and showed that there are some important comorbidities that were significantly associated with in-hospital mortality and other clinical factors that independently influenced mortality in critically ill patients with COVID-19, which may help to categorize these patients and these factors as high-risk and might improve the outcome.

## 5. Conclusions

Patients with COVID-19 requiring ICU admission have a high mortality rate. Underlying comorbidities increase the risk of mortality in these vulnerable patients. In our cohort, the baseline comorbidities that were significantly associated with in-hospital mortality were diabetes, underlying cardiac disease, and underlying kidney disease. Factors that independently influenced mortality in our critically ill patients with COVID-19 were high aPTT, high INR, acidosis, and a high ferritin level.

## Figures and Tables

**Table 1:** Baseline patient demographic and disease characteristics.

Variable	Estimate
Age (years), mean (SD)	52.8 (16.6)
Male sex, *n* (%)	184 (80.4)
Saudi nationality, *n* (%)	59 (25.8)
Body mass index, mean (SD)	29.1 (13.9)
Contact with COVID-19, *n* (%)	80 (34.9)
Recent travel *, *n* (%)	5 (2.2)
Comorbidities, *n* (%)	
Diabetes mellitus	122 (53.3)
Hypertension	106 (46.3)
Renal disease	44 (19.2)
Cardiac disease	59 (25.8)
Respiratory disease	50 (21.8)
Hematology disorder	9 (3.9)
Oncology disorder	7 (3.1)
Postsolid organ transplant	2 (0.9)
CNS disorder	4 (1.8)
HIV infection	2 (0.9)

* Travel to a country with high COVID-19 burden. Abbreviations: CNS, central nervous system; HIV, human immunodeficiency virus; SD, standard deviation.

**Table 2:** Findings that predicted mortality.

Investigation	Survivors (*n* = 145)	Nonsurvivors (*n* = 84)	*p*-Value
Chest radiography, *n* (%)			
Normal	9 (6.3)	3 (3.7)	0.225
Mild consolidation	42 (29.4)	19 (23.2)	
Severe consolidation	66 (46.2)	36 (43.9)	
ARDS	26 (18.2)	24 (29.3)	
ECG abnormality, *n* (%)	57 (54.3)	39 (65.0)	0.180
Low GFR (<90 mL/min/1.73 m^2^), *n* (%)	76 (52.4)	76 (90.5)	<0.001
Hypernatremia, *n* (%)	50 (35.5)	59 (70.2)	<0.001
Hyponatremia, *n* (%)	8 (5.7)	1 (1.2)	0.097
Hyperkalemia, *n* (%)	53 (37.6)	61 (72.6)	<0.001
Anemia, *n* (%)	51 (37.8)	58 (70.7)	<0.001
Leukocytosis, *n* (%)	91 (67.4)	75 (91.5)	<0.001
Leukopenia, *n* (%)	19 (14.1)	18 (22.0)	0.135
Thrombocytopenia, *n* (%)	32 (25.4)	58 (69.1)	<0.001
High aPTT, *n* (%)	92 (71.3)	81 (96.4)	<0.001
High INR, *n* (%)	33 (25.6)	54 (64.3)	<0.001
High D-dimer, *n* (%)	112 (97.4)	84 (100)	0.136
Acidosis, *n* (%)	41 (35.0)	62 (84.9)	<0.001
High troponin, *n* (%)	50 (49.0)	66 (84.6)	<0.001
High ferritin, *n* (%)	32 (27.4)	73 (86.9)	<0.001
High LDH, *n* (%)	126 (96.9)	83 (100)	0.107
Abnormal liver enzyme levels, *n* (%)	98 (73.7)	62 (75.6)	0.753

Abbreviations: AKI, acute kidney injury; aPTT, activated partial thromboplastin time; ARDS, adult respiratory distress syndrome; ECG, electrocardiographic; GFR, glomerular filtration rate; INR, international normalized ratio; KDIGO, Kidney Disease Improving Global Outcome; LDH, lactic acid dehydrogenase.

**Table 3:** Factors that independently predict in-hospital mortality.

Characteristics	Crude Estimate		Adjusted Estimate	
	OR (95% CI)	*p*-Value	OR (95% CI)	*p*-Value
Age (year)	1.02 (1.00, 1.04)	0.049	1.00 (0.97, 1.04)	0.888
Sex	0.75 (0.38, 1.45)	0.390	0.74 (0.20, 2.79)	0.655
Diabetes	1.74 (1.01, 3.01)	0.047	0.82 (0.20, 3.33)	0.786
Cardiac disease	2.69 (1.47, 4.94)	0.001	1.41 (0.38, 5.21)	0.608
Renal disease	3.57 (1.80, 7.06)	<0.001	1.08 (0.24, 4.91)	0.921
Low GFR *	8.63 (3.88, 19.16)	<0.001	0.90 (0.17, 4.77)	0.904
Hypernatremia	4.30 (2.40, 7.68)	<0.001	1.16 (0.32, 4.23)	0.825
Hyperkalemia	4.40 (2.45, 7.93)	<0.001	2.67 (0.73, 9.74)	0.136
Anemia	3.98 (2.21, 7.18)	<0.001	2.31 (0.63, 8.48)	0.206
Leukocytosis	5.18 (2.21, 12.17)	<0.001	3.98 (0.86, 18.3)	0.076
Thrombocytopenia	6.55 (3.55, 12.09)	<0.001	1.36 (0.39, 4.72)	0.628
High APTT	10.86 (3.23, 36.6)	<0.001	7.44 (1.15, 47.9)	0.035
High INR	5.24 (2.88, 9.51)	<0.001	4.10 (1.20, 13.8)	0.025
Acidosis	10.45 (4.96, 22.0)	<0.001	3.75 (1.01, 13.8)	0.047
High Troponin	5.72 (2.76, 11.84)	<0.001	1.85 (0.40, 8.47)	0.428
High Ferritin	17.63 (8.30, 37.4)	<0.001	14.4 (4.17, 49.9)	<0.001

Abbreviations: GFR, glomerular filtration rate; APTT, activated partial thromboplastin time; INR, international normalized ratio. * Low GFR <90 mL/min/1.73 m^2^.

## Data Availability

The data presented in this study are available on request from the corresponding author.
